# Association between waterpipe use and susceptibility to cigarette smoking among adolescents and young adults who never smoked: A systematic review and meta-analysis

**DOI:** 10.18332/tid/159621

**Published:** 2023-02-21

**Authors:** Zhu Yu, Meng Wang, Junfen Fu

**Affiliations:** Department of Endocrinology, Children’s Hospital Zhejiang University School of Medicine, Hangzhou, China; Department of Non-Communicable Diseases Control and Prevention, Zhejiang Provincial Center for Disease Control and Prevention, Hangzhou, China

**Keywords:** waterpipe, cigarettes, smoking susceptibility, meta-analysis

## Abstract

**INTRODUCTION:**

Several factors associated with susceptibility to cigarette smoking have been identified, yet there is little evidence on the possible effects of waterpipe use. With this systematic review and meta-analysis, we aimed to investigate the relationship between waterpipe use and cigarette smoking susceptibility among adolescents and young adults who never smoked.

**METHODS:**

Eligible studies were searched in PubMed, Springer Link, ScienceDirect and Cochrane Library up to August 2022. The pooled odds ratio (OR) and 95% confidence intervals (CI) of cigarette smoking susceptibility with waterpipe use were estimated using a random-effects model. Publication bias was assessed by Egger’s regression asymmetry test and Begg’s rank correlation test with Begg’s funnel plot.

**RESULTS:**

A total of 59710 participants, including 3559 waterpipe users from six studies, were identified in this analysis. Results showed that the odds of susceptibility to cigarette smoking were nearly two times (OR=1.90; 95% CI: 1.59–2.26) greater for never smoker adolescents and young adults who used waterpipe tobacco, compared to those who were never users. In subgroup analyses, the pooled OR was 2.19 (95% CI: 1.52–3.14) and 1.70 (95% CI: 1.51–1.92) for current and ever use of waterpipe, while the pooled OR was 1.99 (95% CI: 1.35–2.95) and 1.87 (95% CI: 1.45–2.39) in Arab and non-Arab nations, respectively.

**CONCLUSIONS:**

Our findings suggest that waterpipe use was associated with greater odds of susceptibility to cigarette smoking among adolescents and young adults who never smoked. Tailored public health policies and regulations on waterpipe smoking may help to protect youth never smokers from initiation of cigarette smoking.

## INTRODUCTION

The tobacco epidemic remains one of the biggest public health challenges the world has ever faced. Although the prevalence of smoking tobacco use has decreased significantly worldwide since 1990^1^, the disease burden is substantial due to tobacco-attributable morbidity and mortality. As estimated by the Global Burden of Disease Study, smoking tobacco use accounted for approximately 7.69 million deaths and 200 million disability-adjusted life-years around the world in 2019^2^. Thus, the persistent concerns emphasizing tobacco control initiatives across nations are still imperative. Given that most smokers began to smoke during adolescence or in early adulthood^3^, the identification of never smoker youth who are at risk of cigarette smoking initiation should be a priority to curb the global tobacco epidemic.

Susceptibility to smoking is defined as the absence of a firm commitment against smoking among never smokers in the future, which has been recognized as a strong predictor of subsequent smoking initiation among younger generations^4-6^. To better target the appropriate health education programs on smoking initiation within the vulnerable populations, it helps to understand more factors related to susceptibility to smoking. At present, multiple studies have suggested that susceptibility to smoking among youth is affected by a series of socioeconomic, environmental and behavioral determinants, including male sex, older age, receiving pocket money, as well as a family member or friends smoking, exposure to secondhand smoking and pro-tobacco advertisements, bullied involvement, and electronic cigarettes use^7-10^. By contrast, there is little evidence on this issue regarding the re-emergence of waterpipe smoking among youth never smokers.

Waterpipe tobacco smoking is a form of tobacco consumption that utilizes a single or multi-stemmed instrument to smoke flavored or non-flavored tobacco, where smoke is designed to pass through water or other liquid before reaching the smoker. Since the introduction of flavored tobacco and the development of the Internet in the 1990s, this centuries-old tobacco use method has been rapidly spreading among youths globally^11,12^. For example, a systematic review showed that the prevalence range of current waterpipe smoking among school students was 12–15% and 9–16% across the USA and the Arabic Gulf region, while for university students, the prevalence was 10% and 6%, respectively^13^. Waterpipe smoking has been a growing public health concern due to accumulating reports of adverse effects on respiratory diseases, cancers, metabolic syndrome, cardiovascular disease, and mental health^14,15^. It is noteworthy that in some populations with an established culture of waterpipe smoking, the age of initiation for waterpipe tends to be earlier than for cigarettes^16^, indicating that waterpipe use may serve as a gateway to cigarette smoking. Several longitudinal studies further supported the gateway theory, finding that waterpipe smoking was associated with later life cigarette smoking initiation^17-20^. However, these studies were concerned with smoking initiation, and it is necessary to consider the earlier phase of susceptibility to smoking. Besides, in light of the widespread perception of waterpipe smoking as being less harmful among users^21^, more evidence is urgently needed to evaluate the possible effects of waterpipe use on cigarette smoking possibilities. We systematically retrieved the existing literature and conducted a meta-analysis to examine the association between waterpipe use and susceptibility to cigarette smoking among never smoker adolescents and young adults.

## METHODS

### Search strategy

The present study followed the Meta-analysis of Observational Studies in Epidemiology (MOOSE) guidelines^22^. Relevant literature focusing on the association between waterpipe use and susceptibility to cigarette smoking were searched on four databases (PubMed, Springer Link, ScienceDirect and Cochrane Library) up to August 2022. No language limit was set in literature searching, but only articles published in English were finally included in this study. Susceptibility to cigarette smoking was defined as the lack of a firm commitment not to smoke among never smokers, without the answer ‘definitely not’ to two or three of the following questions: ‘Do you think you will smoke a cigarette soon?’, ‘Do you think you will smoke a cigarette in the next year (or five years)?’, and ‘If one of your best friends were to offer you a cigarette, would you smoke it?’. The leading search terms of exposure factor included: ‘waterpipe’, ‘shisha’, ‘hookah’, ‘narghile’, ‘arghila’, ‘risk factor’, ‘determinant’, and ‘predictor’, and were screened with Boolean OR. The study outcome was screened by searching keywords for: ‘susceptibility to smoking’, ‘smoking intention’, ‘openness to smoke’, and ‘willingness to smoke’. The exposure and outcome searching items were then linked using Boolean AND. The specific search strategy is presented in Supplementary file Table 1. Reference lists of retrieved literature were also screened.

### Eligibility criteria

Selected studies in this meta-analysis met the following eligibility criteria: 1) investigating the association between waterpipe use and susceptibility to cigarette smoking; 2) providing the effect value with a 95% confidence interval (CI) or data to calculate these; and 3) only within the never smokers.

### Data extraction

Two authors independently assessed the eligibility of studies and extracted information from each eligible study. The information included: 1) the name of the first author; 2) the year of publication; 3) participants and sample size; 4) data source and location; 5) the study design; 6) measures definition; and 7) confounders adjusted. The quality of the included studies was assessed using a modified version of the Newcastle-Ottawa Scale for cross-sectional studies^23^.

### Statistical analysis

Q-test and the I^2^ statistic were used to assess the heterogeneity across studies^24^. For the Q-test, p<0.05 was considered statistically significant. The low, moderate, and high degrees of heterogeneity correspond to I^2^ values of 25%, 50%, and 75%, respectively. If there was significant heterogeneity, a random-effects model would be used to assign the weight of each study according to the DerSimonian and Laird method^25^. If there was evidence of no heterogeneity, we used a fixed-effects model with effect estimates given equal weight to the inverse variance of the study. To test the robustness of the present meta-analysis result, sensitivity analysis was performed by excluding the outliers. Publication bias was assessed by both Egger’s regression asymmetry test and Begg’s rank correlation test (p<0.05 was considered statistically significant) with Begg’s funnel plot. STATA Version 11 software (Stata Corp LP, College Station, TX, USA) was employed to conduct all these statistical analyses.

## RESULTS

### Study screening procedure and characteristics

**Figure 1:**
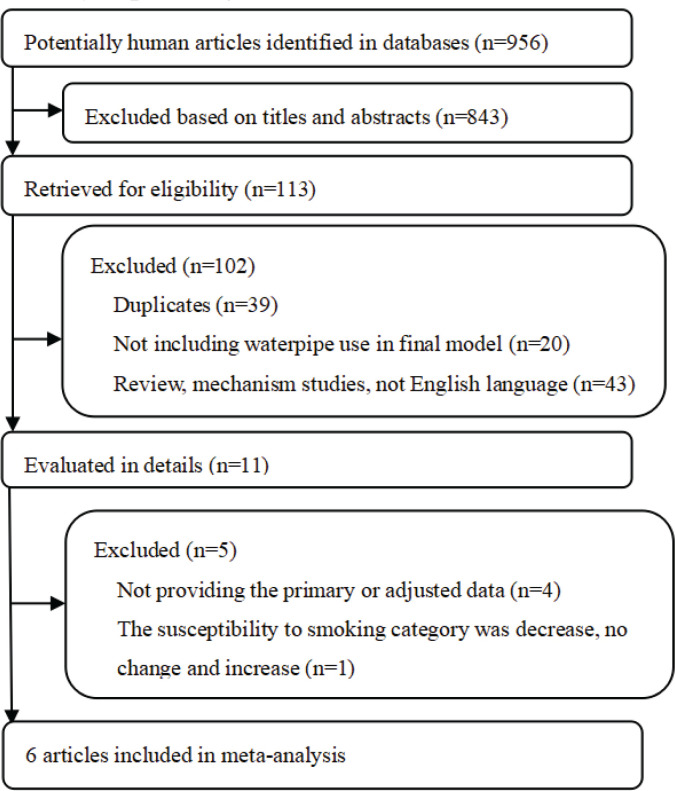
Selection of studies for inclusion in meta-analysis through searching PubMed, Springer Link, ScienceDirect and Cochrane Library, up to August 2022

The flowchart of the study selection is shown in Figure 1. After retrieving the relevant databases, 956 potentially human articles were identified. Then, based on the titles and abstracts, 843 articles were excluded. Among the remaining 113 articles retrieved for eligibility, 102 were excluded because they were duplicates, did not include the exposure factor of waterpipe use, they were review and mechanism studies, and were not written in English. From the remaining 11 articles, four articles did not provide the primary or adjusted data, and 1 article had an inappropriate study outcome, and thus were also excluded. Finally, 6 articles^26-31^ were included in our meta-analysis, of studies conducted in Arab countries, USA and China. Detailed information on included studies is shown in Table 1. Notably, in the publication of Salloum et al.^29^, results were respectively shown for current and ever use of waterpipe, and therefore were considered in the pooled analysis independently. All studies reported the final estimates with adjustments for specific confounders. The quality score of studies ranged from 5 stars to 8 stars, according to the Newcastle-Ottawa Scale (Supplementary file Table 2).

**Table 1:** Information of the six studies included in the meta-analysis

*Authors and Year*	*Source*	*Participants*	*Location*	*Design*	*Measures*	*Variables adjusted*
Veeranki et al.^26^ 2015	2002–2011 Global Youth Tobacco Surveys (GYTS)	School-attending adolescents aged 13–15 years Cases/total: 1471/30711	17 Arab countries including Djibouti, Egypt, Gaza strip, Jordan, Kuwait, Lebanon, Libya, Morocco, Oman, Qatar, Saudi Arabia, Sudan, Syria, Tunisia, United Arab Emirates, West Bank and Yemen	Cross-sectional	Susceptibility to smoking: an adolescent was defined as being susceptible to cigarette smoking based on his or her response on a 5-point ordinal scale ranging from ‘definitely not’ to ‘definitely yes’ to the three following questions: 1) ‘If one of your friends offered you a cigarette, would you smoke it?’; 2) ‘At any time during the next 12 months, do you think you will smoke a cigarette?’; and 3) ‘Do you think you will be smoking cigarettes 5 years from now?’. An adolescent who responded ‘definitely not’ to all three questions was defined as not susceptible, and those who reported any other response to the three questions was defined as being susceptible to cigarette smoking. Waterpipe current use: an adolescent was defined as currently using waterpipe when he or she responded >0 days to the question: ‘During the past 30 days (one month), on how many days did you smoke shisha, hookah, narghile, arghila, or waterpipe?’.	Parental or peer smoking, secondhand smoke exposure inside or outside home, knowledge about harmful effects of smoking and secondhand smoke, exposure to tobacco industry promotions, receptivity of anti-smoking education in schools, country, year of survey administration
Coleman et al.^27^ 2015	2012–2013 National Adult Tobacco Survey (NATS)	Young adults aged 18–29 years Cases/total: 1096/4310	USA	Cross-sectional	Openness to smoking: ‘Do you think you will smoke a cigarette soon?’ and ‘Do you think you will smoke a cigarette in the next year?’. Response options were: ‘Definitely yes’, ‘Probably yes’, ‘Probably not’, and ‘Definitely not’. A binary composite variable was created, and those who responded with any response option other than a firm intention not to smoke (‘Definitely not’) were categorized as being open to smoking cigarettes. Ever use of hookah: ‘Have you ever smoked tobacco in a hookah in your entire life?’. Respondents who selected ‘yes’ were considered to have used hookah at some point in their lifetime.	Sex, age group, race/ethnicity, education level, US Census region, ever use of smokeless tobacco, ever use of electronic cigarettes, ever use of cigars, and ever experimentation with cigarettes
Kheirallah et al.^28^ 2015	2009 Jordan GYTS	School-attending adolescents aged 13–15 years Cases/total: 209/1476	Jordan	Cross-sectional	Susceptibility to smoking: among these never smokers, susceptibility was defined according to response to three questions: ‘Do you think you will be smoking cigarettes 5 years from now?’, ‘At any time during the next 12 months do you think you will smoke a cigarette?’, and ‘If one of your best friends offered you a cigarette, would you smoke it?’. Youth who answered ‘definitely not’ to all three questions were defined as not susceptible to cigarette smoking, and all others were defined as susceptible. Waterpipe ever use: ‘Did you smoke waterpipe?’, youth who answered ‘yes’ or ‘yes, but I stopped now’ were defined as ever waterpipe smokers and those who answered ‘no’ as nonusers.	Sex, age, perceived family affluence, peer smoking, parental smoking and school clustering effect
Salloum et al.^29^ 2016	2012–2013 NATS	Young adults aged 18–24 years Cases/total: 97/2528 for current smokers and 301/2528 for ever smokers	USA	Cross-sectional	Susceptibility to smoking: ‘Do you think you will smoke a cigarette soon?’ and ‘Do you think you will smoke a cigarette in the next year?’. Response options were ‘definitely yes’, ‘probably yes’, ‘probably not’ and ‘definitely not’. Those who responded with ‘definitely not’ to both questions were considered not susceptible to cigarette smoking, whereas all other participants were considered susceptible. Waterpipe (current and ever) use: ‘The next question asks you about smoking tobacco in a hookah. A hookah is a type of water pipe […]. Do you now smoke tobacco in a hookah every day, some days, rarely or not at all?’. Participants who responded with either ‘every day’ or ‘some days’ were defined as current waterpipe smokers. Participants who selected ‘rarely’ were defined as intermittent (i.e. ever) waterpipe smokers. Those who chose ‘not at all’ were defined as non-smokers of waterpipe.	Sex, age, race, education level, annual household income, cigar smoking, smokeless tobacco use, electronic cigarettes use, harmful perception for cigarettes and experimentation with cigarettes
Jiang et al.^30^ 2017	2012–2013 Hong Kong Schoolbased Survey on Smoking among Students	Primary (grade 4–6) and secondary (grade 7–12) school students (mean age: 14.8 years) Cases/total: 18/4479	China	Cross-sectional	Susceptibility to smoking: ‘Do you think you will be smoking cigarettes 12 months from now?’ and ‘If one of your best friends were to offer you a cigarette, would you smoke it?’ on a 4-point Likert scale from ‘definitely no’ to ‘definitely yes’. Those who answered ‘definitely no’ to both questions were considered not susceptible to cigarette smoking and otherwise as susceptible to smoking. Waterpipe current use: ‘In the past 30 days, which of the following products have you used: cigarettes; electronic cigarettes; waterpipe; chewing tobacco; cigars; snus; smoking pipe or snuff; other tobacco product?’. Students who checked ‘waterpipe’ were defined as current waterpipe smokers.	Age, sex, peer cigarette smoking, and living with a cigarette smoker
Bahelah et al.^31^ 2017	2014 National Youth Tobacco Survey (NYTS)	Middle (6–8 grades) and high (9–12 grades) school students from public and private schools Cases/total: 367/16206	USA	Cross-sectional	Susceptibility to smoking: ‘Do you think you will smoke a cigarette in the next year?’, ‘Do you think that you will try a cigarette soon?’, and ‘If one of your best friends were to offer you a cigarette, would you smoke it?’. Each question has four response options: ‘Definitely yes’, ‘Probably yes’, ‘Probably not’, and ‘Definitely not’. Students who answered ‘Definitely not’ to all three questions were considered not susceptible to cigarette smoking and those who gave a response other than ‘Definitely not’ to any of the three questions were considered susceptible to cigarette smoking. Waterpipe current use was defined as smoking waterpipe at least once in the past 30 days.	Sex, race, use of other combustible and non-combustible tobacco products, receptivity to tobacco marketing, beliefs and attitudes towards tobacco use, and exposure to pro-tobacco ads

### Meta-analysis of the association between waterpipe use and susceptibility to cigarette smoking among never smokers

**Figure 2:**
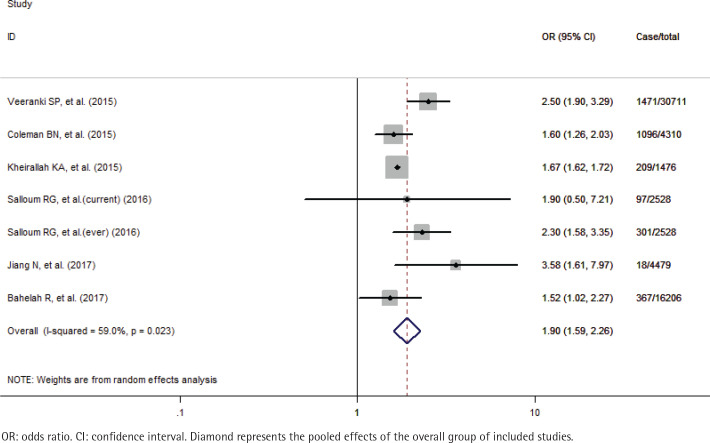
Effects of waterpipe use on susceptibility to cigarette smoking among never smoker adolescents and young adults

**Figure 3:**
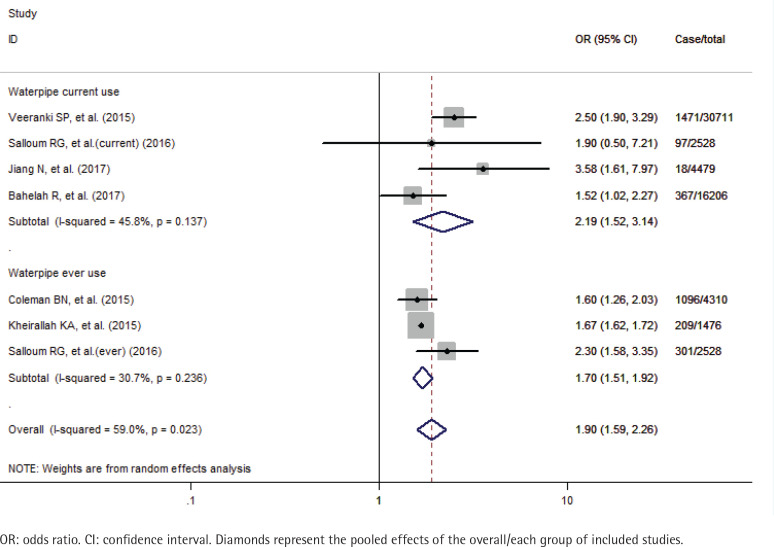
Effects of waterpipe use on susceptibility to cigarette smoking among never smoker adolescents and young adults stratified by status of waterpipe use (current and ever)

**Figure 4:**
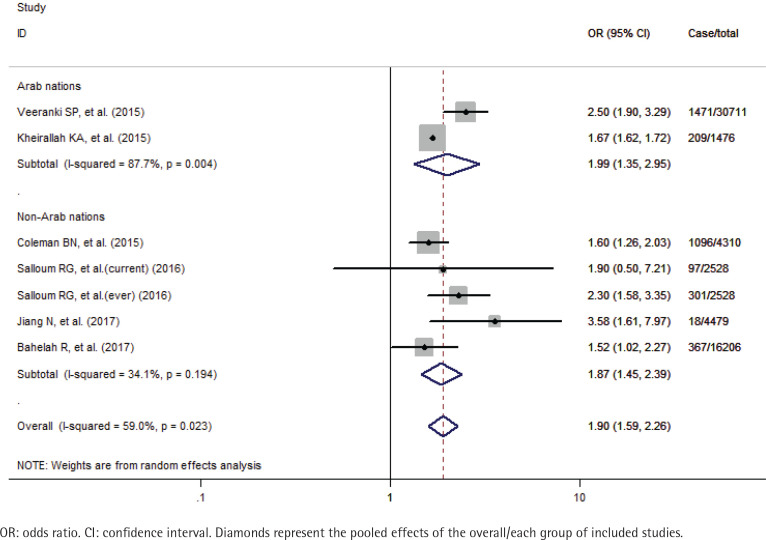
Effects of waterpipe use on susceptibility to cigarette smoking among never smoker adolescents and young adults stratified by study location (Arab and non-Arab countries)

A total of 59710 study participants, including 3559 never smokers who used waterpipe, were identified in this meta-analysis. A formal test for heterogeneity gave a significant result (p=0.023, I^2^=59.0%), and a random-effects model was used to calculate the odds ratio (OR) of susceptibility to cigarette smoking with waterpipe use. The pooled analysis showed that never smoker adolescents and young adults who used waterpipe had greater odds of susceptibility to cigarette smoking (OR=1.90; 95% CI: 1.59–2.26) than those who never used it (Figure 2). The sensitivity analysis results showed that the studies of Veeranki et al.^26^ and Jiang et al.^30^, substantially influenced the final pooled estimate in the current study. After excluding the two studies, the OR was 1.67 (95% CI: 1.62–1.72) without any between-study heterogeneity (p=0.535, I^2^=0%). Besides we also performed the subgroup analyses by the status of waterpipe use (current and ever) and study location (Arab and non-Arab countries). Specifically, for current and ever use of waterpipe, the pooled OR was 2.19 (95% CI: 1.52–3.14) and 1.70 (95% CI: 1.51–1.92), respectively (Figure 3); for studies in Arab and non-Arab countries, the pooled OR was 1.99 (95% CI: 1.35–2.95) and 1.87 (95% CI: 1.45–2.39), respectively (Figure 4).

### Publication bias

**Figure 5:**
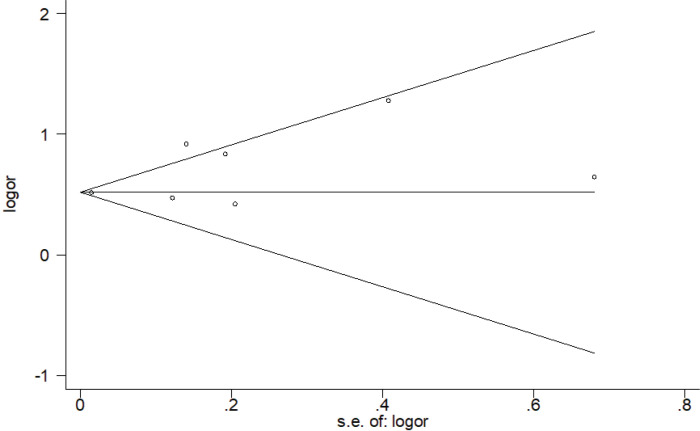
Begg’s funnel plot (with pseudo 95% confidence intervals) to detect any publication bias

Regarding the assessment of publication bias, neither Egger’s regression asymmetry test (p=0.151) nor Begg’s rank correlation test (p=0.548) gave a statistically significant result. Besides, Begg’s funnel plot showed no striking evidence of publication bias (Figure 5).

## DISCUSSION

Several studies have demonstrated a positive relationship between waterpipe use and subsequent cigarette smoking among young people. Yet, scientific evidence on their linkage is not well-established and commonly focused on those who have already initiated smoking^17-20^. Given that susceptibility to smoking is the precursor and strong predictor of future established smoking, to tailor interventions targeted at the initiation of smoking among youth never smokers, it is helpful to extend this knowledge to the association with waterpipe use. To our knowledge, this meta-analysis study is probably the first to synthesize the existing literature to investigate the possible effects of waterpipe use on cigarette smoking susceptibility among never smoker adolescents and young adults.

Overall, the pooled estimate shows that never smoker adolescents and young adults who used waterpipe have nearly two times greater odds of susceptibility to cigarette smoking, after adjustment for potential confounders. Considering the differences in sample size, study populations characteristics and confounders adjustment within the included studies, a sensitivity analysis is simultaneously performed. After analysis, the result is observed to be essentially unchanged, strengthening our conclusions with a small number of studies. These findings provide supportive evidence that waterpipe use may act as a gateway to cigarette smoking initiation among youth never smokers.

Although possible mechanisms linking the waterpipe use behavior to the initiation of cigarette smoking are not yet fully understood, it is hypothesized that the nicotine exposure and dependence caused by waterpipe smoking probably play a contributory role in the proposed gateway effect. For one thing, laboratory research has suggested that waterpipe users are exposed to a similar or even higher level of nicotine relative to cigarettes, which could lead to high nicotine dependence^32,33^. Mainly, as adolescents are susceptible to nicotine with the developing brain^34^, younger users are prone to develop nicotine dependence and may use tobacco products later to avoid withdrawal symptoms or cravings^35,36^. For another, according to the most recent data from the Global Youth Tobacco Survey, secondhand smoke exposure is still a serious global health issue in adolescents^37^ and supposed to be an essential source of nicotine exposure. Prior evidence has demonstrated that secondhand smoke is significantly related to nicotine dependence and initiation of cigarette smoking among non-smokers^38^. Specific to waterpipe, although the exhaled smoke has been drawn through the water, epidemiological studies based on real-world settings consistently found a markedly high concentration of airborne nicotine in the waterpipe venues and cafés^39,40^.

In addition, to explore the possible source of the moderate degree of heterogeneity across studies and its mediating effects imposed on the association between waterpipe use and cigarette smoking susceptibility, we perform the subgroup analyses stratified by status of waterpipe use (current and ever) and study location (Arab and non-Arab countries). The analysis results confirm the significant positive associations with low evidence of heterogeneity in most subgroups. Interestingly, the reported ORs are much greater for current waterpipe use (2.19 vs 1.70) and Arab countries (1.99 vs 1.87) than their counterparts. These observed stronger effects, in a way, are consistent with previous reports that nicotine in the inhaled smoke increased with the intensity of waterpipe smoking^41,42^, and the prevalence of waterpipe use was significantly higher in youths of Arab origin^43^. Based on these findings, we propose that the characteristics of use patterns and cultural background of waterpipe smoking should be considered in further studies on the linkage between waterpipe use and cigarette smoking susceptibility.

### Strengths and limitations

The present study has several strengths. This is the first meta-analysis study investigating the effects of waterpipe use on susceptibility to cigarette smoking among never smoker youths with strict eligibility criteria. Besides, the larger number of total participants and waterpipe users included in the analysis may be another strength.

There are some limitations that should also be considered. First, our meta-analysis study only includes studies written in English by searching for limited available databases; however, no significant publication bias is detected. Second, the betweenstudy heterogeneity is moderate in the main analysis and still exists in the subgroup analysis by the status of waterpipe use and study location, which suggests that other unexplored factors for the variation may be present. Third, as all the included studies are cross-sectional, the causality of the association between waterpipe use and cigarette smoking susceptibility cannot be inferred from the current results, and more well-designed prospective studies focused on the never smoker population are warranted.

## CONCLUSIONS

Findings from this meta-analysis study indicate that waterpipe use is positively associated with greater odds of susceptibility to cigarette smoking among never smoker adolescents and young adults. To protect never smokers from initiation of cigarette smoking, more tailored public health policies and regulations specific to waterpipe smoking are of major importance and should be urgently developed.

## Data Availability

The data supporting this research are available from the authors on reasonable request.
