# Supplementary material for: Association between waterpipe use and susceptibility to cigarette smoking among adolescents and young adults who never smoked: A systematic review and meta-analysis
Source: Tob Induc Dis. 2023 Feb 21;21:29. doi: 10.18332/tid/159621 (PMC9942264; doi:10.18332/tid/159621)
Supplement: Supplementary file 1 [file TID-21-29-s1.pdf]

Supplementaty Table 1. Specific searching strategies for databases.

Database	Searching strategies
Pubmed	((((susceptibility to smoking[Title/Abstract]) OR (smoking intention[Title/Abstract])) OR (openness to smoke[Title/Abstract])) OR (willingness to smoke[Title/Abstract])) AND ((((((((waterpipe[Title/Abstract]) OR (shisha[Title/Abstract])) OR (hookah[Title/Abstract])) OR (narghile[Title/Abstract])) OR (arghila[Title/Abstract])) OR (risk factor[Title/Abstract])) OR (determinant[Title/Abstract])) OR (predictor[Title/Abstract])) (n=60)
Springer Link	((susceptibility to smoking) OR(smoking intention) OR (openness to smoke) OR (willingness to smoke)) AND ((waterpipe OR shisha OR hookah OR narghile OR arghila OR (risk factor) OR determinant OR predictor)) (n=68)
ScienceDirect	(susceptibility to smoking OR smoking intention OR openness to smoke OR willingness to smoke) AND (waterpipe) (n=229);<br>(susceptibility to smoking OR smoking intention OR openness to smoke OR willingness to smoke) AND (shisha) (n=82);<br>(susceptibility to smoking OR smoking intention OR openness to smoke OR willingness to smoke) AND (hookah) (n=395);<br>(susceptibility to smoking OR smoking intention OR openness to smoke OR willingness to smoke) AND (narghile) (n=81);<br>(susceptibility to smoking OR smoking intention OR openness to smoke OR willingness to smoke) AND (arghila) (n=2).
Cochrane Library	((susceptibility to smoking) OR(smoking intention) OR (openness to smoke) OR (willingness to smoke)) AND ((waterpipe OR shisha OR hookah OR narghile OR arghila OR (risk factor) OR determinant OR predictor)) [Title/Abstract/keyword]<br>(n=39)

Supplementary Table 2. Newcastle-Ottawa Scale adapted for cross-sectional studies.

	Veeranki SP, et al.	Coleman BN, et al.	Kheirallah KA, et al.	Salloum RG, et al.	Jiang N, et al.	Bahelah R, et al.
Selection: (Maximum 5 stars)						
(1)Representativeness of the sample:						
a) Truly representative of the average in the target population. * (all subjects or random sampling)	★	★	★	★		★
b)Somewhat representative of the average in the target population. * (non-random sampling)					★	
(2)Sample size:						
a) Justified and satisfactory. *	★	★	★	★	★	★
(3)Non-respondents:						
a) Comparability between respondents and non-respondents characteristics is established, and the response rate is satisfactory. *	★	★	★	★		★
(4)Ascertainment of the exposure (risk factor):						
a) Validated measurement tool. **	★★		★★			
b) Non-validated measurement tool, but the tool is available or described.*		★		★		★
Comparability: (Maximum 2 stars)						
(1)The subjects in different outcome groups are comparable, based on the study design or analysis. Confounding factors are controlled.						

a) The study controls for the most important factor (select one). *						
b) The study control for any additional factor. *	★	★	★	★	★	★
Outcome: (Maximum 3 stars)						
(1) Assessment of the outcome:						
a) Independent blind assessment. **						
b) Record linkage. **						
c) Self report. *	★	★	★	★	★	★
(2)Statistical test:						
a) The statistical test used to analyze the data is clearly described and appropriate, and the measurement of the association is presented, including confidence intervals and the probability level (p value). *	★	★	★	★	★	★

© 2023 Yu Z. et al.
